# Vibration Serviceability of Footbridges: Classical vs. Innovative Material Solutions for Deck Slabs

**DOI:** 10.3390/ma13133009

**Published:** 2020-07-06

**Authors:** Izabela Joanna Drygala, Joanna Maria Dulinska, Rafał Ciura, Kamil Lachawiec

**Affiliations:** Faculty of Civil Engineering, Cracow University of Technology, 31-155 Kraków, Poland; jdulinsk@pk.edu.pl (J.M.D.); ciura.er@gmail.com (R.C.); kamill.lachawiec@gmail.com (K.L.)

**Keywords:** footbridges, composite material, numerical modeling, innovative applications, dynamic analysis, vibration comfort criteria assessment, glass fiber-reinforced polymer (GFRP)

## Abstract

In this study, the human-induced dynamic performance of modern footbridges equipped with either classical reinforced concrete (RC) or innovative glass fiber-reinforced polymer (GFRP) composite deck slabs were investigated and compared. The numerical studies were carried out for two bridges: a three-span cable-stayed footbridge and a three-span continuous beam structure. Two variants of both bridges were taken into consideration: the footbridges equipped with traditional RC slabs and the structures benefitted with GFRP slabs. The risk of resonance as well as the vibration serviceability and the comfort criteria assessment of the footbridges with different slab materials were assessed. The investigation revealed that the footbridges, both cable-stayed and beam, benefitted with the GFRP slabs had higher fundamental frequency than those with the traditional RC slabs. The footbridges with the GFRP slabs were less exposed to the resonance risk, having fundamental frequencies above the limit of the high risk of resonance. The effect of shifting up the natural frequencies by introducing GFRP slabs was more remarkable for the lightweight beam structure than for the cable-stayed footbridge and resulted in a more significant reduction of the resonance risk. The calculated maximum human-induced accelerations of the footbridges benefitted with the GFRP slabs were meaningfully higher than those obtained for the footbridges with the RC slabs. The study proved that, with the same GFRP slab, meeting vibration serviceability and comfort criteria limits in the case of very lightweight beam structures may be more problematic than for cable-stayed footbridges with more massive structural systems. In the research, particular attention was paid to examining the impact of higher harmonics of the moving pedestrian force on the structures benefitted with the GFRP composite slabs. It occurred that in the case of footbridges, both cable-stayed and beam, equipped with the RC slabs higher harmonics of human force did not play any role in the dynamic performance of structures. However, in the case of the footbridges benefitted with the GFRP slabs, the impact of higher harmonics of the pedestrian force on the dynamic behavior of structures was clearly visible. Higher harmonics excited accelerations comparable to those executed by the first harmonic component. This conclusion is of great importance for footbridges equipped with GFRP slabs. The fundamental frequency may place a footbridge in the low or even negligible risk resonance range and the higher frequencies corresponding to vertical modes may be located above the limit of 5 Hz that ensures avoiding resonance. Nevertheless, the fact that fundamental modes are so responsive to higher harmonics significantly increases the risk of resonance. The amplification of the dynamic response may occur due to frequencies related to second or third harmonics (i.e., being half or a third of the natural frequencies). In such cases, full dynamic analysis of a footbridge at the design stage seems to be of crucial importance.

## 1. Introduction

The use of fiber-reinforced polymer (FRP) composite materials in bridge engineering has grown significantly over the past decades. The advantages of FRPs over traditional construction materials are demonstrated in their low density, which results in reduced mass as well as high strength and stiffness-to-weight ratios. Comparative case studies have shown that the average FRP composite footbridge may be even half the weight of a steel footbridge, with the same performance, and it is five times lighter than its concrete counterpart [1]. The advantage of low weight results in reduced costs in transportation, assembly, and foundation works. There are substantial benefits in terms of durability as FRP composites show high resistance to corrosion what makes them a rational choice also in terms of low maintenance costs [2]. Moreover, FRP material is resistant to de-icing chemicals and has also good fire resistant properties. 

However, attracted by superior mechanical and aesthetical properties, like slenderness and lightweight of innovative FRP footbridges, bridge engineers must not forget about the serviceability limits and comfort criteria. Slimline and esthetical footbridges can also be relatively flexible, which renders them more susceptible to pedestrian induced vibration than structures made of traditional materials. Moreover, it seems that nowadays people are becoming more sensitive to vibrations that could be partly a reaction to increasing environmental influences [3]. Therefore, progress in FRP footbridges requires further research, especially the development of new guidelines. Currently, designers of FRP footbridges use standards for structures made of traditional materials, especially in the field of dynamics and vibration serviceability of FRP footbridges. Pre-standards and guidelines that have appeared in recent years mainly relate to statics or basic structural dynamics [4,5,6,7].

The most recent state-of-art research on FRP footbridge dynamics is presented in [8]. The authors measured dynamic properties and considered experimental works of other authors of a large set of FRP as well as conventional pedestrian bridges (138 objects in total). The comprehensive summary of dynamic properties of footbridges served for a critical comparison of innovative FRP structures against structures made of conventional materials. 

Nowadays, the innovative composite materials are used not only for building all-FRP footbridges. A lot of pedestrian bridges which are FRP deck only structures are being designed and erected as well [9,10]. Composite decks provide greater durability under aggressive environments [11] and reduced maintenance costs over the life of the footbridges [12]. They are a good alternative to heavy popular concrete decks, due to their lightweight, high strength, and anticorrosion properties [13]. They may be assembled using adhesive bonding or mechanical interlocking, making the construction process easy and reducing installation times compared to conventional steel or concrete solutions [14]. However, FRP decks of footbridges offering architecturally attractive solutions show some disadvantages as far as their mechanical properties are concerned. The relatively thin external FRP walls of panels make them prone to concentrated out-of-plane loads (e.g., high heels impact, umbrella ferrules, and dropped tools) [15,16]. The experiments on the GFRP decks indicated that even relatively mild impact events could result in damages [17].

The research on the dynamics of footbridges with FRP decks are presented in works [18,19,20]. Modal characteristics investigation, dynamic performance under human activity evaluation as well as vibration serviceability assessment of two footbridges with FRP decks are provided in the paper [18]. The two structures have different structural systems (suspension and simple beam) resulting in distinctly different natural frequencies. Six vibration modes were identified for the suspension bridge in the frequency range up to 5 Hz, while there was one mode only for the simple beam bridge. A comparison of the vibration behavior under human-induced excitation, however, revealed that they exhibit similar responsiveness to impact forces of excitation. Generally, FRP footbridges are recognized to be more responsive to human-induced dynamic loading than conventional structures. It is stated that the maximum accelerations of FRP footbridges can be even 3.5 times higher than those of conventional footbridges of similar geometry [8]. However, according to vibration serviceability design guidelines for footbridges [21], excessive vibration will not occur if a footbridge has a fundamental vertical frequency above 5 Hz. This limit ensures avoiding resonance excitation by the first two walking harmonics. As FRP footbridges are so sensitive to pedestrian-induced dynamic loading, high probability appears that their modes could be responsive to excitation by higher harmonics of the moving force. The authors of [8] conclude that for these reasons the minimum frequency limit suitable for conventional footbridges might be not appropriate for FRP footbridges. 

The main objective of this paper is to compare and to assess the dynamic performance of footbridges benefitted with innovative glass fiber-reinforced polymer (GFRP) deck slabs with those equipped with classical reinforced concrete (RC) slabs. To accomplish the objective, the human-induced dynamic responses of two footbridges equipped with either traditional (RC) or innovative (GFRP) deck slabs were investigated. Especially, the risk of resonance, the vibration serviceability and the comfort criteria meeting of the footbridges with different deck materials were assessed. 

The research on the dynamic performance of footbridges with GFRP decks were carried out for two pedestrian bridges erected recently in Poland: a three-span cable-stayed footbridge and a three-span continuous beam footbridge. The existing objects are equipped with RC deck slabs; however, at the design stage, the possibility of benefiting the objects with innovative GFRP decks was taken into account. Therefore, in this study, two variants of both footbridges were taken into consideration: first, their decks were equipped with existing traditional RC slabs, and second, the decks were benefitted with hypothetical innovative GFRP slabs.

Two aspects were taken into account in the selection of the objects. The first aspect addressed architectural reasons. The use of GFRP slabs leads to a noticeable reduction in thickness of the deck slab which makes the design slimmer and more eye-friendly. Innovative materials implementation in landmark facilities makes them even more unique and interesting. The existed cable-stayed footbridge, being attractive from the architectural point of view, has become a classical landmark. In case of such a sophisticated bridge shape it could be interesting to take design up to the next level, using innovative GFRP composite material, which has a lot to offer in terms of the aesthetical appearance of a footbridge. The footbridge benefited with a GFRP slab, much thinner than a RC slab, can give the desired impression of lightness, slenderness, and uniqueness. In turn, the beam footbridge, being a simple low-budget design, can be easily replicated. Having a well-adjusted design of GFRP decks, it would be possible to apply new composite materials on a large scale. 

The second aspect of the object selection concerned structural reasons. The footbridges were selected so that the change of the deck slab material from RC to GFRP affected the masses of the objects, and thus their dynamic characteristics, in significantly different ways. In the case of the cable-stayed footbridge with a complex structural system consisting of many members, changing the slab material from RC to GFRP will not cause a significant difference in its total mass. In turn, introducing the same changes to the beam footbridge with a simple structural system leads to remarkable mass reduction. Therefore, qualitative changes in dynamic performance resulting from replacing an RC slab with a GFRP slab can be similar for these two selected footbridges, whereas quantitative changes may occur dissimilar enough to result in different conclusions on the possibility of meeting vibration serviceability and comfort criteria limits.

The novelty of the research lies in performing comprehensive numerical studies on the dynamic performance of two real footbridges equipped either with existing RC deck slabs or with hypothetical GFRP deck slabs. As the structural systems of the footbridges remained unchanged, the differences in the dynamic behavior resulted only from replacing classical RC slabs with innovative GFRP ones. Moreover, the assessment of changes in the dynamic performance, resulted from replacing RC with the GFRP slabs, provided for two footbridges which differ much in the structural systems, also makes this study innovative in the field of bridge dynamics. This aspect is not highlighted in other studies. 

Finally, taking into account the fact that FRP footbridges are more responsive to human-induced dynamic loading than conventional structures, higher harmonics of forces generated during walking or running could play a significant role in the dynamic performance of a footbridge. In the study, what is also a novelty of the research, particular attention was paid to examining the possibility of exciting substantial accelerations of pedestrian bridges by higher harmonics of walking or running force in case of structures benefitted with innovative composite deck materials. This problem of serviceability is rarely addressed in the context of the rational and balanced reply to the question of whether to use innovative or classical materials for decks of footbridges.

## 2. Materials and Methods

### 2.1. Risk of Resonance and Comfort Criteria Assessment for Footbridges

The main purpose of the dynamic investigation of footbridges is the assessment of resonance risk and their vibration comfort criteria since human susceptibility to vibrations is very high. People, who are the major receivers of the dynamic behavior of footbridges, may feel unpleasant or even may not tolerate vibrations that exceed a certain level of accelerations. 

The methodology of assessing the resonance risk is provided by the SETRA publication [21]. According to these guidelines, the ranges of natural frequencies of footbridges in vertical and horizontal directions associated with particular levels of resonance risk are summarized in Table 1. The limits are valid for footbridges with natural frequencies less than 5 Hz. The guidelines for design practice are also presented in Eurocodes and other standards [22,23,24]. 

Comfort criteria, recommending rules for the assessment of the impact of vibrations on users of footbridges, are widely studied in the literature [24,25,26,27]. The practical rules for comfort criteria assessment are provided by Eurocodes standards [24]. According to this document, verification of the comfort criteria should be performed if the fundamental frequency of the footbridge is less than 5 Hz in a vertical direction or less than 2.5 Hz in a horizontal direction. The acceptable level of vertical accelerations is 0.7 m/s^2^. Practical guidelines for the comfort criteria assessment of footbridges are proposed in the SÉTRA document [21]. The acceleration ranges in the vertical and horizontal directions with comfort levels assigned are presented in Table 2. 

### 2.2. Dynamic Loading Generated during Human Movement 

Forces generated by the pedestrians may be dangerous for the construction of footbridges, especially if they can induce the resonance phenomenon. The most predictable kind of pedestrians’ motions are running and walking. Both of them are periodic and change in time and space. The ranges of frequency typical for human moving are summarized in Table 3.

For the footbridges with the risk of resonance, the dynamic response of the structure to human movement has to be evaluated and assessed. It requires an appropriate mathematical model of forces generated by pedestrians walking or running. In recent studies, there are a lot of theoretical models of loading induced by a single pedestrian activity on a footbridge [21,27,28,29,30,31,32]. 

The loading generated by a single pedestrian moving (walking or running) can be modeled by a periodic function *F*(*t*), which may be resolved into a Fourier series. The sum of all unitary components of this function returns the total effect of the periodic action. In this study, the loading generated by a single pedestrian moving is exhibited as a sum of dynamic and static parts by the formula [21,33]

(1) $$F\left(t\right)=G\left[1+\overset{n}{\underset{i=1}{\sum }}A_{i}sin(2i\pi f_{s}t-\phi_{i})\right],\;\;\;\;i=1,\;2,\ldots ,\;n$$

where *F*(*t*)—the loading generated by a single pedestrian moving; G—the weight of pedestrian; $f_{s}$—the fundamental loading frequency; $A_{i}$ and $\phi_{i}$—the amplitude and the phase angle of the i-th harmonic, respectively; and *n*—the number of harmonics taken into consideration.

The Fourier coefficients $A_{i}$ and $\phi_{i}$, appearing in Equation (1), are called Dynamic Load Factors (DLF). They can be referenced to a board literature review and their adoption is based on experimental research. For the purposes of this study, the DLFs occurring in Equation (1) for a different kind of motion are shown in Table 4. Such values, originally reported by [33,34], are nowadays discussed and used by many researchers [28,30,35]. 

In the case of walking, it is strongly recommended [21] that the minimum of three harmonics in the Fourier series must be taken into consideration to find the typical “saddle” shape of the single pedestrian walking periodic function. 

The sine model applied in Equation (1) is adequate for the walking case, because each step overlaps with the previous one what makes the force continuous. However, for the running pedestrian, this assumption seems to be not fully acceptable, as the generated loading has discontinuous nature. The dynamic force estimated with Equation (1) is negative in the time interval corresponding approximately to the flight phase during the running. Hence, negative values must not be taken into account. In the case of running, the phase shifts are assumed to be negligible [21]. 

### 2.3. Structural Layouts and FE (finite element) Models of the Footbridges with Traditional RC Slabs

#### 2.3.1. Geometry and Material Data of the Three-Span Cable-Stayed Footbridge

**Figure 1:**
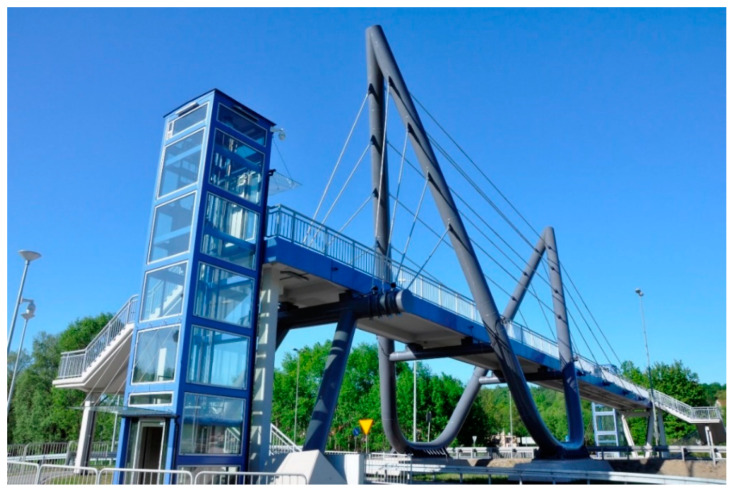
The general view of the three-span cable-stayed footbridge.

The first footbridge investigated in this research was a three-span cable-stayed footbridge located in Southern Poland which serves to carry pedestrians above the national expressway. The structure was erected in 2017 and it was designed according to Polish national standards [36]. The general view of the structure is shown in Figure 1. 

**Figure 2:**
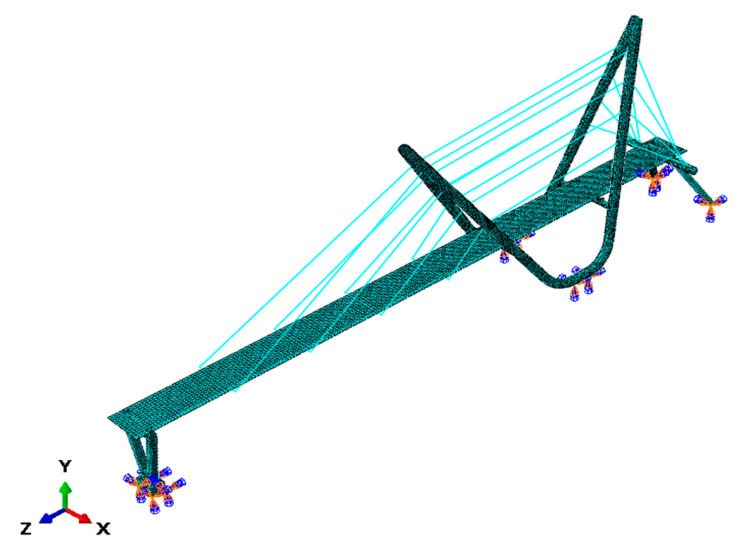
The FE (finite element) model of the three-span cable-stayed footbridge.

**Figure 3:**
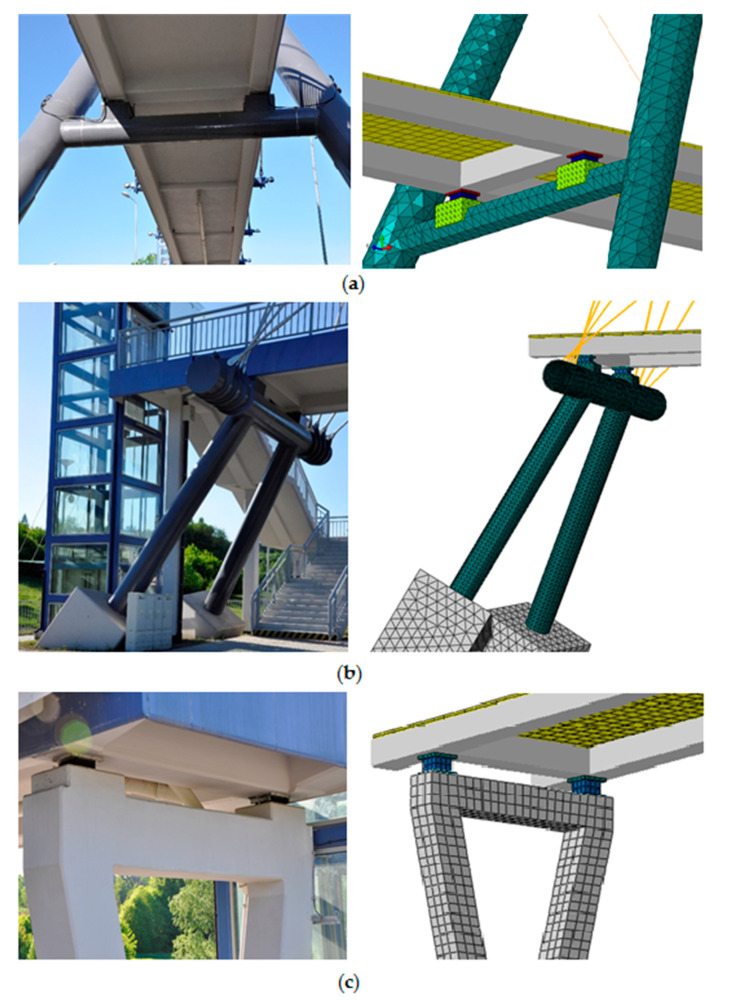
The structural details of the footbridge and the FE model: (**a**) the pylon arms tied by horizontal pipe with the crossbar supporting the deck; (**b**) the footbridge deck located on the steel beam anchoring the suspension strings; (**c**) the reinforced concrete V-shaped support.

The three-dimensional FE model of the cable-stayed footbridge was created using the ABAQUS/Standard software (Dassault Systemes, Velizy-Villacoublay, France) [37]. The whole model of the structure is shown in Figure 2, whereas the structural details and their implementation in the FE model are presented in Figure 3.

The total length of the structure is 52.36 m. The structural system of the footbridge is composed of a superstructure attached to the steel pylon with steel cables as linking elements. 

The pylon is built of two symmetrically inclined pipes with a wall thickness of 40 mm, which form the shape of a letter “V”. At the deck level, the pylon arms are tied by horizontal pipes with a wall thickness of 30 mm, which support the deck (Figure 3a). The cables suspending the deck structure and the cables connecting the pylon arms are attached to the upper part of the pylons (above the footbridge deck). To increase the rigidity of the structure, the pylon was partly filled with concrete. In the FE model, the upper empty parts of pylons and the crossbars of pipe cross section were modeled with linear triangular shell elements of type S3. Quadratic solid elements of type C3D10 were used to reflect the shape and properties of the concrete-filled parts of the pylons.

The superstructure of the bridge consists of two reinforced concrete girders with a rectangular cross section of 400 × 480 mm, four reinforced concrete diaphragms with a rectangular cross-section of 500 × 400 mm and four steel crossbars with a tubular cross section with a diameter of 137 mm and a thickness of 25 mm. The reinforced concrete slab of the deck is 3.4 m wide and 0.255 m thick. Linear beam elements of type B31 were used to model the superstructure, whereas linear quadrilateral shell elements of type S4R were applied to model the slab.

The footbridge deck is located on a steel beam also anchoring the suspension strings (see Figure 3b), on the crossbars of pylons and on a reinforced concrete support in the shape of a closed “V” (see Figure 3c). The steel supporting beam was modeled with shell elements of type S3, whereas for the “V” support solid elements C3D10 were used. 

The elastic truss elements (T3D2) with no compressive stiffness (“no compression” option) were used for modeling hangers and cables to guarantee that compressive stresses would not be generated during dynamic analysis [37]. However, when such a numerical approach is used, instability of the model can appear. This difficulty was overcome by overlaying each truss element, which has no compression stiffness, with beam elements with low compression stiffness (5% of the cables’ stiffness). This enables obtaining stiffness greater than zero, which has the effect of stabilizing the model. “Tie” constraints were used to guarantee identical kinematic behavior of the truss and the beam elements.

The structure is founded on three reinforced concrete block foundations. However, the high rigidity of the footbridge subsoil means that all foundations, being merged with the ground, do not play any role in the dynamic behavior of the structure. Therefore, the foundations were not implemented in the FE model and the fixed boundary conditions were imposed at all structure to foundations connection points (see Figure 2). 

For numerical analysis the modulus of elasticity of steel parts of the structure was assumed as 205 GPa. The Poisson’s ratio was taken as 0.30. The mass density of 7850 kg/m^3^ was adopted. The material data of concrete were assumed: the modulus of elasticity—30 GPa, the Poisson’s ratio—0.20, and the mass density—2400 kg/m^3^. 

The footbridge deck is benefitted with elastomeric bearings which link the superstructure and the supports. The bearings were modeled as three solid elements of type C3D8R corresponding to two outer steel sheets and an elastomer in between. The middle bearing layer was modeled as a material of average elastomer and steel properties based on sets formed from 8 cm elastomer layers and 3 cm steel inserts. The two-parameter Mooney–Rivlin model of elastomer material (assumed as C10 = 0.292 MPa and C01 = 0.177 MPa) was replaced with the equivalent elasticity modulus (2.814 MPa). Such simplification is often used in calculations of bridges with elastomeric bearings [38]. The Poisson’s ratio of elastomeric material was taken as 0.49. 

#### 2.3.2. Geometry and Material Data of the Three-Span Continuous Beam Footbridge

The second structure investigated in this research is a bicycle–pedestrian footbridge, which is now under construction in Cracow, Southern Poland. It will serve to carry pedestrians and cyclists above the national expressway. The total length of the footbridge is 80 m. The structural system of the object is designed as a three-span continuous beam. 

**Figure 4:**
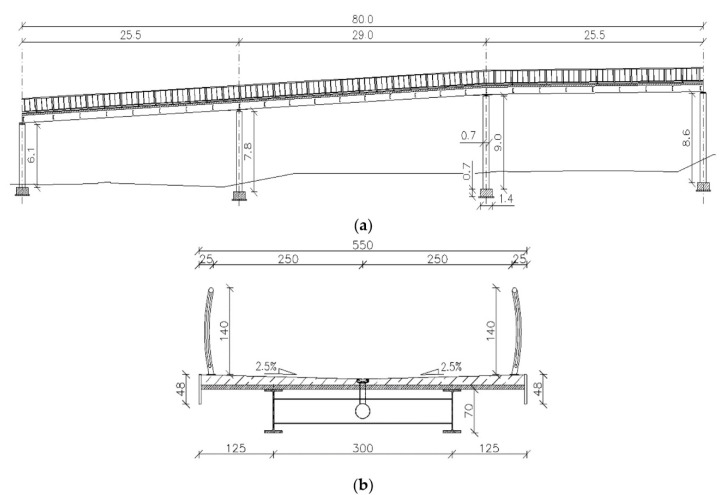
The general view with dimensions [m] (**a**) and the cross-section with dimensions [cm] (**b**) of the three-span continuous beam footbridge with the reinforced concrete (RC) slab.

The three-dimensional FE model of the continuous beam footbridge was created using the ABAQUS/Standard software (Dassault Systemes, Velizy-Villacoublay, France) [37]. The longitudinal view of the footbridge is shown in Figure 4a, whereas the cross section of the beam is shown in Figure 4b.

**Figure 5:**
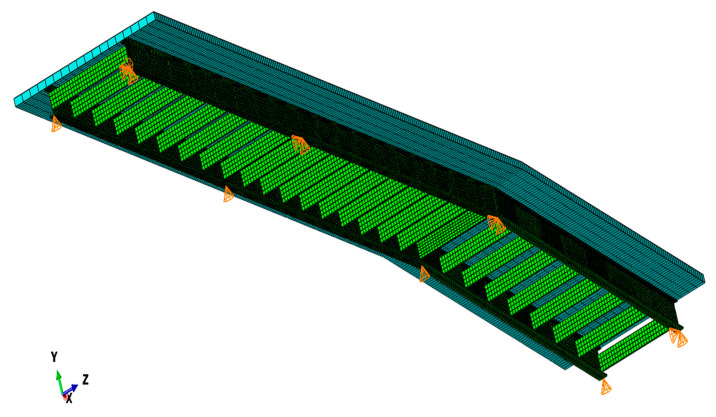
The FE model of the three-span continuous beam footbridge.

In this research, only the superstructure of the footbridge was modeled and investigated. The primary structural system of the footbridge consists of two steel girders HEB700 shaped as H-beams located at a distance of 3 m. The height of the H-beams equals 0.7 m, whereas the width of the beams is 0.3 m. The thickness of the H-beams’ upper and bottom flange as well as for the web are 32 and 17 mm, respectively. The main girders are connected by steel C-beam crossbars C400 which are 0.4 m high and 0.11 m wide with the 18 mm thick upper and bottom flange and 14 mm thick web. These members made a structural system working as a grid. The upper flanges of the steel girders are integrated with the reinforced concrete slab. The slab is 5.5 m wide and 0.2 m thick. The grid and the slab were modeled with bilinear quadrilateral shell elements of type S8R. The FE model of the three-span continuous beam footbridge with boundary conditions and the FE mesh is presented in Figure 5. Accordingly to the construction design of the footbridge, the modulus of elasticity of steel parts of the structure was assumed as 205 GPa, the Poisson’s ratio equaled 0.30 and the mass density of 7850 kg/m^3^ was adopted. The material data of reinforced concrete were assumed: the modulus of elasticity—33 GPa, the Poisson’s ratio—0.20, and the mass density—2600 kg/m^3^. 

### 2.4. Structural Layouts and FE Models of the Footbridges with Innovative GFRP Slabs

In the second stage of this study, the vibration serviceability of the footbridges with slabs made of innovative glass fiber-reinforced polymers (GFRP) was examined. New variants of models of the investigated footbridges, having GFRP instead of RC slabs, were created. The structural layouts of the footbridges and the main dimensions of all members of both footbridges remained unchanged. Only the deck slabs were changed in terms of both, material and dimensions.

**Figure 6:**
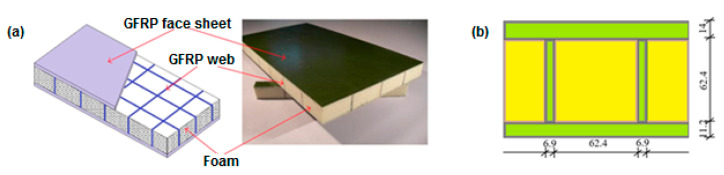
The innovative glass fiber-reinforced polymers (GFRP) composite sandwich slab: (**a**) the structure of the slab [40]; (**b**) the 3D model and the dimensions of the adopted composite slab [mm].

Most kinds of FRP decks can be classified into the modular type or the sandwich-type [39,40]. For this study purposes, a typical sandwich footbridge deck consisted of GFRP face sheets and foam-web core has been taken into consideration. The sample of the composite material of the sandwich slab with its structure [40] is shown in Figure 6a. The 3D numerical model and the dimensions of the composite slab, adopted for this study, are shown in Figure 6b. The total thickness of the GFRP composite slab was 87.6 mm in the case of both footbridges.

The outer composite structural part of the slab is built of 0.7 mm thick layers of a different angle of fiber configuration. In the FE model shell elements were applied to the composite material. This approach makes it possible to adopt the classical lamination theory (CLT) [41]. The main assumption of the CLT is that there exists a perfect bond between all laminas in composite laminates. The parameters adopted for the composite material were discussed in detail in several works on a prototype of FRP footbridge [42,43,44]. The mechanical properties adopted for this study are as follows [44]: elasticity modulus in the fiber direction—$E_{1}$= 23.40 GPa; elasticity modulus in the direction perpendicular to the fibers—$E_{2}$= 7.78 GPa; shear moduli—$G_{12}=3.52\;\mathrm{GPa},\;G_{13}=G_{23}=2.30\;\mathrm{GPa}$; Poisson‘s ratio—$\nu_{12}$ = 0.153. The mass density of the composite material was assumed as 1600 kg/m^3^. The parameters of the foam, which is the inner part of the slab material, are assumed as follows: the modulus of elasticity—200 MPa, the Poisson’s ratio—0.30, and the mass density 100 kg/m^3^. 

**Figure 7:**
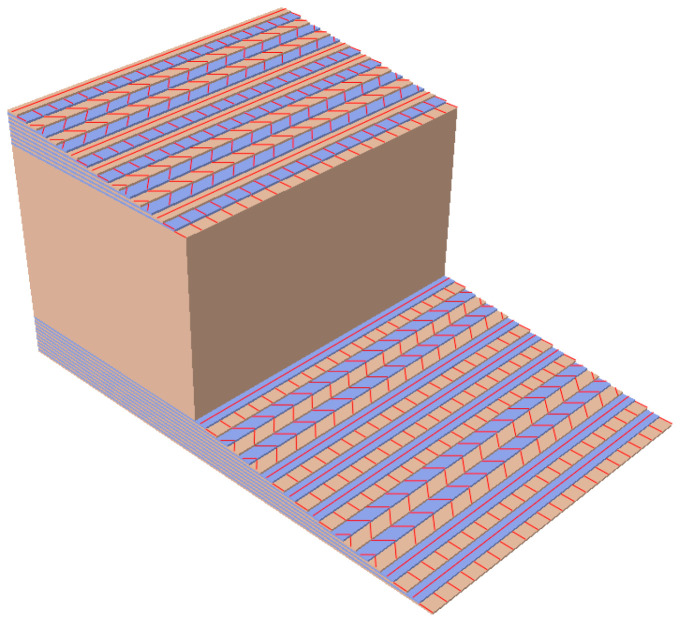
The FE model of GFRP composite sandwich slab with plies of different fiber orientation.

The FE model of the sandwich GFRP slab was prepared with the Abaqus model of a shell composite layup [37]. In this model, conventional shell elements that discretize the reference surface of each ply are used. Conventional shell composite layups were composed of plies made of different materials in different orientations (rotation angle). For each ply the material, thickness, and orientation were specified. In Table 5 particular plies used for constructing the sandwich GFRP composite slabs, adopted on the bases of the Abaqus standard model of the composite layup, are summarized. The top flange was made of 20 laminate layers, and the bottom flange was made of 16 layers. The FE model of the composite slab along with the graphical representation of all plies and their different fiber orientation is shown in Figure 7.

## 3. Results

### 3.1. Natural Frequencies and Modes of Vibration of the Footbridges 

**Figure 8:**
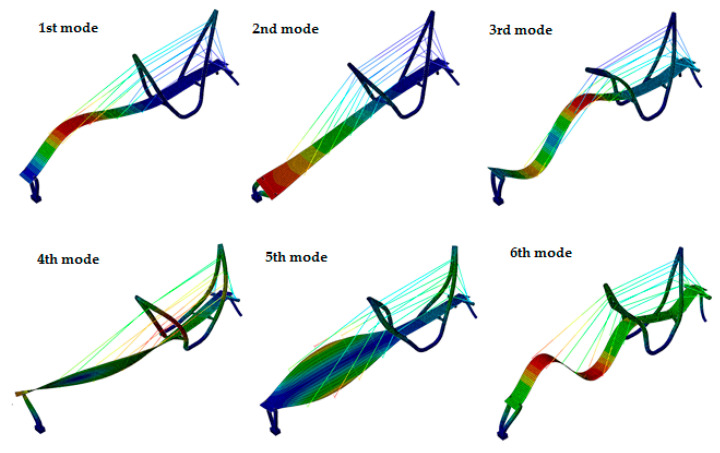
The mode shapes of the cable-stayed footbridge with the GFRP slab.

**Figure 9:**
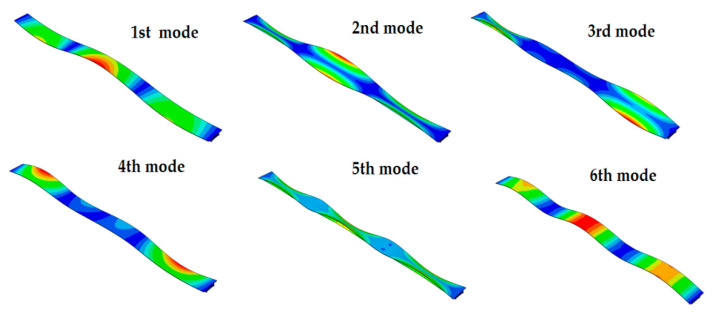
The mode shapes of the beam footbridge with the GFRP slab.

The mode shapes of the cable-stayed and the beam footbridges, equipped with the GFRP slab, are presented in Figure 8 and Figure 9, respectively. Similar mode shapes were obtained for the structures with the RC slabs, but their sequence has changed.

The frequencies corresponding to the vertical mode shapes, substantial for dynamic performance under human movement, obtained for both variants of slab material are compared in Table 6. It is clearly visible from Table 6 that the natural frequencies of the beam structure are mostly lower than that those of the cable-stayed structure. Due to this fact the beam bridge is potentially more exposed to the resonance risk than the cable-stayed one. The study also proved that the frequencies calculated for the structures with GFRP slabs are generally greater than those obtained for the RC slab. 

### 3.2. The Strategy of Pacing Frequency Selection of a Single Pedestrian Moving

Based on the natural frequencies obtained for both footbridges, the strategy of the pacing frequency selection was adopted. Particular modes of vibrations of footbridges could respond to excitation not only by the first harmonic of the walking force appearing in Equation (1), but by higher harmonics as well. Due to this fact, it is reasonable to consider walking or running frequencies related to footbridge dynamic properties, i.e., frequencies at which subsequent harmonics excite subsequent vertical modes of vibrations. Therefore, to evaluate the dynamic response of the footbridges to a single pedestrian moving and to assess their comfort criteria the strategy of the selection of the pacing frequency $f_{s}$, applied in Equation (1) was adopted as follows. Firstly, pacing frequencies typical for a single pedestrian slow, normal and fast walking and running (see Table 3) were taken into account. Second, walking or running frequencies related to the dynamic properties of the footbridges were considered. In particular, the frequencies at which three subsequent harmonics (i.e., 1st, 2nd, and 3rd harmonics) excited up to the 3rd vertical mode of the structure were assumed for calculations. Obviously, only frequencies located within pedestrian dynamic influences ranges, i.e., possible to be generated by a human movement, were taken into consideration. The analysis revealed that the slow and normal human walking/running, in terms of the pacing frequency, excited meaningfully lower accelerations than the fast moving. Therefore, the dynamic responses of the footbridges to the slow and normal walking/running are not presented in the paper. 

### 3.3. Damping Ratios Adopted for the Footbridges with the RC and GFRP Slabs

In the structural dynamic performance assessment the damping ratio selection is of a crucial meaning and, if possible, should be adopted experimentally. In this study, the damping ratio of 1.83% was assumed for the footbridges with RC slabs. Such value was obtained through the in situ tests of the investigated cable-stayed footbridge [45]. For both footbridges with the GFRP slabs the damping ratio was assumed on the basis of literature. A comprehensive study of dynamic properties of eight FRP footbridges [8] shows that these structures have usually higher damping ratios, especially for the first vertical mode, than those made of traditional materials. The damping ratios of the investigated FRP footbridges are located in the range from 0.4 to 7.9% with the mean value of 2.5%. Authors investigated two suspension footbridges with FRP decks (Wilcott Bridge, 53.1 m long and Halgavor Bridge, 47 m long) [8,46]. The Wilcott Bridge was reported to have a damping ratio of 2.5% for the first vertical mode and significantly lower values, from 1.0 to 1.9%, for the higher vertical modes. In the case of Halgavor Bridge, lower damping ratios were recognized: 2.3% for the first vertical mode and 0.3–1.5% for the higher vertical modes. The damping ratios of the Wilcott Bridge was also measured and reported as 1.64 and 0.72% for the second and third vertical modes, respectively [47]. A single-span beam footbridge 16.9 m long was investigated and reported in [18]. It was found that the damping ratio for the first frequency varied with vibration acceleration amplitudes, from 1.1% at 0.01 m/s^2^ to ~1.7% at 0.1 m/s^2^. Finally, the research on damping ratios was conducted for two FRP footbridges: a cable-stayed all-FRP bridge with a main span of 63 m and a two-span cable-stayed FRP laboratory bridge 19 m long, composed of FRP girders and deck, and steel pylons and cable strands [48]. The extremely low damping ratio of 0.4% was measured for the first structure. The damping ratios for particular vertical modes of the second bridge were laying in the range from 0.4 to 1.2%. Taking into consideration the values of damping ratios presented by the cited authors as well as similarity, in terms of the natural frequencies and mode shapes, of the footbridges and the tested structures investigated in this study, a damping ratio of 2.5% for the first vertical mode and 1.0% for the higher modes were adopted for the analyzed footbridges. 

### 3.4. Vibration Performance of the Cable-stayed Footbridge

The dynamic performance of the footbridges was assessed based on the maximum vertical accelerations generated by the single pedestrian moving, obtained from time histories of accelerations at representative points of the structures. For the study, the pedestrian weight applied in Equation (1) was assumed as G = 700 N. The amplitude A_i_ and the phase angle ϕ_i_ of the i-th harmonic of Fourier series, were taken from Table 4 for a single pedestrian walking or running, respectively. 

**Figure 10:**
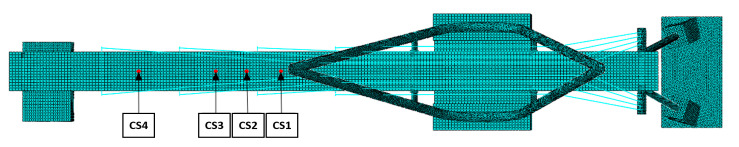
Location of points on the deck of the cable-stayed footbridge selected for the analysis of dynamic performance under human movement: CS1, CS2, CS3, and CS4.

Four representative points located on the footbridge deck were selected for assessing the vibration performance of the cable-stayed footbridge (Figure 10). At points CS4, CS2, and CS1 extremal amplitudes of the 1st, the 2nd, and the 3rd vertical modes occurred, respectively. Point CS3 was located in the middle of the longer footbridge span. 

#### 3.4.1. The Cable-stayed Footbridge Exposed to a Single Pedestrian Walking

The maximum accelerations generated by a single pedestrian walking obtained at the representative points of the cable-stayed footbridge with RC slab for the selected frequencies are presented in Table 7. The maximum value of 0.15 m/s^2^ was obtained at point CS4 for the 1st natural frequency 2.40 Hz. It means that the accelerance peak appeared for the 1st mode and was excited by the 1st harmonics. The investigated higher harmonics did not excite comparable accelerations, and therefore they did not play any role in the dynamic behavior of the structure.

The dynamic responses of the analyzed footbridge, which is originally equipped in the RC slab, to different pedestrian activities were experimentally tested [45]. On the basis of the experimental results, the calculated maximum acceleration was verified. For estimation of the response of the footbridge to dynamic loading generated by a group of people, the multiplication factor *m* is introduced [49]. A perfectly synchronous action by more than one person in comparison to the action of one person results in an increase which is nearly proportional to the number *n* of the persons involved (*m* = *n*). In case of a totally random action by more than one person, no synchronization of pedestrians action is detected and the multiplication factor $m=\sqrt{n}$ is considered appropriate. Through the carried out experiment the accelerations generated by the totally random walking of a group of nine pedestrians at the frequency 2.2 Hz were recorded. The maximum acceleration obtained from the experiment was 0.13 m/s^2^ and it occurred at point CS4. The accelerance peak at point CS4 due to a single pedestrian walking at the frequency of 2.2 Hz obtained numerically was 0.043 m/s^2^. In the case of the group of nine people random walk the multiplication factor *m =* 3. Therefore, the calculated maximum acceleration generated by one pedestrian and the registered maximum acceleration generated by nine people random walk were found to be in perfect agreement.

Then, the maximum accelerations generated by a single pedestrian walking obtained for the cable-stayed footbridge with the GFRP slab are summarized in Table 8. The 1st natural frequency corresponding with the 1st vertical mode (3.45 Hz) is located outside the frequency range of a single pedestrian walking. However, the analysis revealed that the higher harmonics excited relatively high accelerations. The maximum value of 0.21 and 0.17 m/s^2^ appeared at point CS4 and CS3, respectively, at the frequency of 1.73 Hz. It means that the 2nd harmonics strongly excited the 1st vertical mode. Then, at points CS1 and CS2 the maximum accelerations of similar values were obtained at the frequency of 2.06 Hz. Therefore, at these points, the 3rd harmonics excited the 2nd vertical mode causing the maximum accelerations.

#### 3.4.2. The Cable-stayed Footbridge Exposed to a Single Pedestrian Running

The maximum accelerations, obtained at the frequencies related to the dynamic properties of the cable-stayed footbridge with the RC slab, generated by a single pedestrian running are presented in Table 9. The maximum acceleration of 0.36 m/s^2^ occurred at point CS4 for the 1st natural frequency of 2.4 Hz. It is clearly visible that the higher harmonics excited significantly lower accelerations.

The dynamic responses of the analyzed cable-stayed footbridge with the RC slab to different types of pedestrian running were also positively verified in the experimental way [45]. The accelerations generated by a single pedestrian running at the frequency of the normal walk 2.7 Hz were recorded. The peak value obtained from the experiment was 0.13 m/s^2^ and it occurred at point CS4. The same result was obtained numerically. 

Then, the maximum accelerations, obtained at the frequencies related to the cable-stayed footbridge with the GFRP slab, generated by a single pedestrian running are shown in Table 10. 

The maximum acceleration of 0.59 m/s^2^ was observed at point CS4 for the frequency of 3.3 Hz. This frequency is close to the 1st natural frequency of 3.45 Hz. Therefore, approximately, the accelerance peak appeared for the 1st mode and was excited by the 1st harmonics. The same situation occurred at point CS3, where the maximum acceleration equaled 0.46 m/s^2^. However, higher harmonics also excited comparable accelerations. The maximum value obtained at point CS1 for the frequency of 3.09 Hz equaled 0.51 m/s^2^. It means that the 2nd harmonics mostly excited the 2nd vertical mode. Then, at point CS2 the maximum acceleration of 0.4 m/s^2^ was obtained for the frequency of 2.06 Hz. Therefore, at this point, the 3rd harmonics strongly excited the 2nd vertical mode. 

### 3.5. Vibration Performance of the Beam Footbridge 

**Figure 11:**
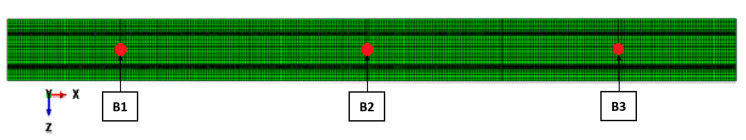
Location of points on the deck of the three-span beam footbridge selected for the analysis of dynamic performance under human movement: B1, B2, and B3.

Three representative points located on the beam footbridge deck were selected for assessing the vibration performance of the beam footbridge (Figure 11). At points B1, B2, and B3, which are located in the middle of each span, extremal amplitudes of the 1st, the 2nd, and the 3rd vertical modes occurred, respectively. 

#### 3.5.1. The Beam Footbridge Exposed to a Single Pedestrian Walking

The maximum accelerations, obtained for the frequencies related to the dynamic properties of the beam footbridge with the RC slab, generated by a single pedestrian walking are presented in Table 11. The maximum value of 0.06 m/s^2^ was obtained at point B2 for the 1st natural frequency of 2.0 Hz. The accelerations excited by other walking frequencies were much lower. Hence, the dynamic performance of the beam footbridge with the RC slab is not affected by the higher harmonics.

Next, the maximum accelerations, obtained at the frequencies selected for the beam footbridge with GFRP slab, generated by a single pedestrian walking are presented in Table 12. The maximum acceleration at all control points equaled 0.26 m/s^2^. It was obtained either at the frequency 1.91 Hz (at which the 2nd harmonics excited the 2nd vertical mode) or at the frequency 1.64 Hz (at which the 3rd harmonics excited the 3rd vertical mode). 

**Figure 12:**
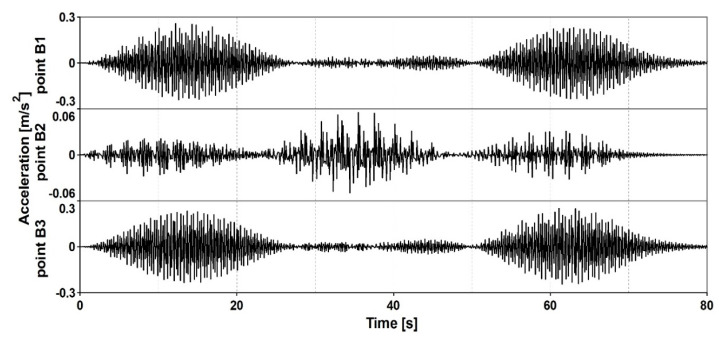
Comparison of time histories of accelerations obtained at the frequency of 1.91 Hz at points B1, B2, and B3.

The detailed comparative analysis of the time histories of accelerations obtained at the frequency of 1.91 Hz at all control points B1, B2, and B3 was carried out (Figure 12). The maximum acceleration of 0.26 m/s^2^ appeared at point B1 located on the first span at the time 12.2 s. At the same moment the amplitude at point B3 located on the third span showed similar value but opposite direction. The acceleration at point B2 located on the middle span was one order lower. This investigation demonstrated that at the frequency of 1.91 Hz (the half of the 2nd frequency 3.82 Hz) the 2nd harmonics of the walking force executed the 2nd mode shape.

#### 3.5.2. The Beam Footbridge Exposed to a Single Pedestrian Running

The maximum accelerations, obtained at the frequencies selected for the beam footbridge with RC slab, generated by a single pedestrian running are presented in Table 13. The maximum value of 0.18 m/s^2^ was obtained at point B2 for the 1st natural frequency of 2.0 Hz. The frequencies related to higher harmonics were beyond the single pedestrian running frequency range. 

Next, the maximum accelerations, obtained for the beam footbridge with the GFRP slab, generated by a single pedestrian running are presented in Table 14. 

The maximum value of 0.48 m/s^2^ was obtained at point B2 at the 1st natural frequency 2.76 Hz. Hence, the accelerance peak appeared for the 1st mode and was excited by the 1st harmonics. However, the 2nd harmonics excited the 2nd vertical mode and generated similar maximum accelerations: the values obtained at points B1 and B3, for the frequency 1.91 Hz, equaled 0.43 and 0.44 m/s^2^, respectively. Moreover, the 2nd harmonics exciting the 3rd mode at the frequency of 2.45 Hz generated similar values of maximum accelerations (up to 0.46 m/s^2^). 

## 4. Discussion—Traditional RC vs. Innovative GFRP Deck Slab 

### 4.1. Risk of Resonance Levels 

The risk of resonance of the footbridges with the RC and the GFRP slab was assessed according to the SÉTRA document [21]. Only the frequency ranges in the vertical directions were taken into consideration in this study. The assessment results for the cable-stayed and the beam footbridge are summarized in Table 15. 

For the 1st, 2nd, and 3rd natural frequencies of the cable-stayed bridge with the RC slab the medium, low and negligible risk of resonance appeared, respectively. After benefitting the structure with the GFRP slab, the natural frequencies were all shifted up. The low risk of resonance appears for the 1st frequency, whereas for the 2nd and 3rd frequencies the risk of resonance is negligible. The results of the resonance risk assessment for the beam footbridge are quite similar. 

The comparative analysis provided in Table 15 proved that the footbridges equipped with the GFRP slabs were less exposed to the risk of resonance than those with the RC slabs. It is also worth noticing that the risk of resonance of the lightweight beam footbridge with the RC slab was assessed as high for the 1st natural frequency of 2.00 Hz. The effect of shifting up the fundamental frequency by introducing the GFRP slab resulted in the noteworthy reduction of the resonance risk.

### 4.2. Vibration Serviceability and Comfort Criteria Assessment of the Footbridges

Vibration serviceability assessment is considered more crucial in the design of GFRP footbridges than in the design of similar structures made of conventional construction materials [8]. To assess the dynamic performance of the cable-stayed and the beam footbridge equipped the traditional RC or the innovative GFRP slab the maximum accelerations were compared in Table 16.

The accelerance peaks of the cables-stayed footbridge with the GFRP slab executed by a single pedestrian walking and running were, respectively, 1.4 and 1.6 times higher than the maximum values calculated for the footbridge with the classical RC slab. Next, the maximum acceleration of the beam footbridge with GFRP was 4.3 times higher than those of the footbridge with the classical RC slab in case of a walking pedestrian and 2.7 times higher in case of a running pedestrian. These results are in good agreement with the statement that the accelerance peaks of FRP footbridges are, on average, 3.5 times higher than those of conventional footbridges [8]. 

The comparisons also revealed that the beam footbridge became more sensitive to vibration excitation by humans being equipped with the GFRP slab than the cable-stayed one. Such an effect occurred due to the relations between the masses of the structures. A total mass as well as modal masses strongly influence the dynamic performance of a structure. The masses of the cable-stayed and the beam footbridge with the RC or the GFRP slab are compared in Table 17.

The total mass of the cable-stayed footbridge with the RC slab was 1.4 times larger than that with the GFRP slab, whereas the same ratio for the beam footbridge equaled 4.2 times. In the case of the beam footbridge, introducing of the GFRP slab resulted in 3 times greater change in its mass than in the case of the cable-stayed one. Similar relations were observed between the modal masses: the RC/GFRP mass ratios are around 2.5 times higher for the beam than for the cable-stayed footbridge. 

Next, the accelerance peaks of the cable-stayed and the beam footbridges with the GFRP decks were 0.59 and 0.48 m/s^2^, respectively. Therefore, the maximum accelerations did not exceed the acceptable level of 0.7 m/s^2^ provided by EC [24]. However, taking into consideration that the excessive vibration could occur even for two people running on the structures with the GFRP slabs, it may be stated that meeting vibration serviceability requirements may be more challenging for the footbridge with the innovative GFRP than with the conventional RC deck. 

Finally, the acceleration comfort criteria of the footbridges with RC and with GFRP slabs were assessed and compared. The acceleration comfort levels of both material variants were qualified accordingly to the levels of vertical acceleration ranges proposed in SÉTRA guidelines (see Table 3). In the case of both footbridges equipped with the RC slabs the level of comfort was assessed as “maximum”. But the cable-stayed footbridge with the GFRP slab exposed to a single pedestrian running exhibited the maximum acceleration of 0.59 m/s^2^, therefore its comfort level was assessed as “mean”. 

### 4.3. Impact of Higher Harmonics of a Human-induced Force on the Dynamic Performance of the Footbridges

The influence of higher harmonics of forces executed by pedestrian activities on the dynamic performance of the footbridges with the RC and the GFRP slabs was also the objective of this study. 

In the case of the footbridges, both cable-stayed and beam, equipped with the RC slabs, it can be seen that the maximum vertical accelerations excited by the pedestrian walking or running were obtained at the 1st natural frequency. The accelerance peaks appeared in the middle of the main spans, therefore the 1st modes were excited. The investigated higher harmonics excited meaningfully lower accelerations, so did not play any role in the dynamic performance of the footbridges.

For both footbridges benefitted with the GFRP slabs, the dynamic performance occurred more complex and the impact of the higher harmonics on the dynamic behavior under pedestrian activities was clearly visible. If the 1st natural frequency was located inside the single pedestrian movement frequency range, the maximum acceleration appeared at this frequency and the 1st mode of vibration was executed. However, the analysis revealed that the 1st vertical mode was also significantly responsive to excitation by 2nd harmonics of the walking force corresponding with the half of the 1st mode. Moreover, it occurred that the higher harmonics also excited relatively high accelerations due to a single pedestrian walking or running. The magnitudes of accelerations excited by the 2nd and the 3rd harmonics were comparable with those obtained at the 1st harmonics. Finally, the investigations proved that the pacing frequency related to a particular natural frequency executed mode shape adequate to this frequency. 

It is worth noticing that the natural frequencies corresponding to the 2nd and 3rd vertical modes the cable-stayed footbridge with GFRP slab, i.e., 6.17 and 7.53 Hz, were located above the limit of 5 Hz. However, they strongly affected the dynamic performance of the footbridge, as it responded to the frequencies related to the 2nd and 3rd harmonics (i.e., frequencies that are half or a third of the natural frequencies). 

## 5. Conclusions

In this study, the human-induced dynamic response of two modern footbridges equipped with either the traditional reinforced concrete (RC) or the innovative (GFRP) composite deck slabs were investigated and compared. The risk of resonance as well as the vibration serviceability and the comfort criteria of the footbridges with different slab materials were assessed. Especially, particular attention was paid to examining the impact of higher harmonics of the walking or running force on the structures benefitted with the GFRP composite slabs. The following conclusions can be formulated on the basis of the comparative analysis of the dynamic performance of footbridges benefitted with different types of deck slabs:

1. The footbridges, both cable-stayed and beam, benefitted with GFRP slabs had higher fundamental frequency than those with traditional RC slabs. Such a phenomenon occurred due to the fact that the mass decrease in the structures with GFRP slabs was larger than the decrease in their stiffness in comparison with the traditional bridges of identical geometry. The footbridges equipped with the GFRP slabs were less exposed to the risk of resonance than those with the RC slabs, as they had fundamental frequencies shifted up, beyond the ranges of the high risk of resonance. The effect of shifting up the natural frequencies by introducing GFRP slabs was more remarkable in the case of the lightweight beam structure than for the cable-stayed footbridge and resulted in a significant reduction of the resonance risk.
2. The calculated maximum accelerations of the footbridges benefitted with the GFRP slabs executed by a single pedestrian moving were meaningfully higher than those obtained for the footbridges with the classical RC slabs. Therefore, meeting vibration serviceability and comfort criteria limits may be more challenging for the footbridges with the GFRP slabs. The study also proved that meeting vibration serviceability and comfort criteria limits in case of very lightweight beam structures with GFRP slabs may be more problematic than for cable-stayed footbridges with GFRP slabs, having more massive structural systems.
3. In the case of footbridges, both cable-stayed and beam, equipped with the RC slabs the higher harmonics of the human force did not play any role in the dynamic performance of structures. However, in the case of the footbridges benefitted with the GFRP slabs, the impact of higher harmonics of the pedestrian force on the dynamic behavior of structures was clearly visible. Higher harmonics excited accelerations of amplitudes comparable to those executed by first harmonic component.

The last conclusion is of great importance for footbridges equipped with GFRP slabs. The fundamental frequency may place a footbridge in the low or even negligible risk resonance range. The consecutive frequencies corresponding to vertical modes may be located above the limit of 5 Hz that ensures avoiding resonance. Nevertheless, the fact that fundamental modes are so responsive to higher harmonics significantly increases the risk of resonance. The amplification of the dynamic response may occur due to frequencies related to second or third harmonics (i.e., being half or a third of the natural frequencies). Therefore, the dynamic analysis seems to be vital in case of footbridges with GFRP deck slabs even though the lowest natural frequencies are above the limit of 5 Hz. Such a situation, where higher harmonics excite amplitudes comparable to those executed by first harmonic component, does not occur in case of footbridges with RC deck slabs. The minimum frequency limit warrants meeting the vibration serviceability requirements in case of these conventional structures.

The presented analysis may be supportive at the design stage of modern pedestrian bridges in the answer to the question of whether traditional materials are still the best solution for deck slabs or innovative composites might be a better choice, regarding vibration serviceability issues. 

## Figures and Tables

**Table 1:** The resonance risk levels and corresponding ranges of natural frequencies [21].

Risk of Resonance	Frequency Ranges [Hz]	
	Vertical	Horizontal
maximum	1.7–2.1	0.5–1.1
medium	1.0–1.7; 2.1–2.6	0.3–0.5; 1.1–1.3
low	2.6–5.0	1.3–2.5
negligible	0–1.0; >5.0	0–0.3; >5.0

**Table 2:** Ranges of comfortable use [21].

Comfort Level	Ranges of Comfort [m/s^2^]	
	Vertical	Horizontal
maximum	0.0–0.5	0.00–0.15
mean	0.5–1.0	0.15–0.30
minimum	1.0–2.5	0.30–0.80
uncomfortable	>2.5	>0.8

**Table 3:** Typical pacing frequencies for walking and running [25].

Type of Movement	The Range of Frequencies [Hz]			
	Total	Slow	Normal	Fast
walking	1.4–2.4	1.4–1.7	1.7–2.2	2.2–2.4
running	1.9–3.3	1.9–2.2	2.2–2.7	2.7–3.3

**Table 4:** Coefficients of Fourier decomposition [28,30,33,34].

Type of Movement	A_1_	$\phi_{1}$ (rad)	A_2_	$\phi_{2}$ (rad)	A_3_	$\phi_{3}$ (rad)
walking [33]	0.4	0	0.1	1.57	0.1	1.57
running [34]	1.6	0	0.7	0	0.2	0

**Table 5:** The ply table in the composite layup.

Upper Sandwich GFRP Layer, 14 mm Thick																				
No. of ply	1	2	3	4	5	6	7	8	9	10	11	12	13	14	15	16	17	18	19	20
Rotation angle [°]	0	90	0	90	−45	45	−45	45	0	90	0	90	0	90	−45	45	−45	45	0	90
**Inner Sandwich FOAM Layer, 62.4 mm Thick**																				
No. of ply	1																			
Rotation angle [°]	0																			
**Bottom Sandwich GFRP Layer, 11.2 mm Thick**																				
No. of ply	1	2	3	4	5	6	7	8	9	10	11	12	13	14	15	16				
Rotation angle [°]	0	90	−45	45	−45	45	0	90	0	90	−45	45	−45	45	0	90				

**Table 6:** Comparison of natural frequencies of the cable-stayed and the beam footbridges with the RC and GFRP slabs, corresponding to basic vertical modes.

Footbridge	Slab Variant	Natural Frequency [Hz] Corresponding to:		
		1st Vertical Mode	2nd Vertical Mode	3rd Vertical Mode
Cable-stayed	RC	2.40	4.45	5.61
	GFRP	3.45	6.17	7.53
Beam	RC	2.00	2.73	7.78
	GFRP	2.76	3.83	4.91

**Table 7:** Maximum vertical accelerations generated by a single pedestrian walking obtained at the representative points of the cable-stayed footbridge with the RC slab for the selected frequencies.

Point	Maximum Accelerations [m/s^2^] at Frequency:			
	2.4 Hz Fast Walking 1st Natural Frequency	2.23 Hz 2nd Harmonics Excites 2nd Mode	1.48 Hz 3rd Harmonics Excites 2nd Mode	1.87 Hz 3rd Harmonics Excites 3rd Mode
CS1	0.09	0.06	0.08	0.04
CS2	0.10	0.06	0.07	0.03
CS3	0.14	0.06	0.08	0.03
CS4	0.15	0.05	0.05	0.03

**Table 8:** Maximum vertical accelerations generated by a single pedestrian walking obtained at the representative points of the cable-stayed footbridge with the GFRP slab for the selected frequencies.

Point	Maximum Accelerations [m/s^2^] at Frequency:		
	2.4 Hz Fast Walking	1.73 Hz 2nd Harmonics Excites 1st Vertical Mode	2.06 Hz 3rd Harmonics Excites 2nd Vertical Mode
CS1	0.11	0.12	0.19
CS2	0.06	0.14	0.17
CS3	0.13	0.17	0.13
CS4	0.12	0.21	0.11

**Table 9:** Maximum vertical accelerations generated by a single pedestrian running obtained at the representative points of the cable-stayed footbridge with the RC slab for the selected frequencies.

Point	Maximum Accelerations [m/s^2^] at Frequency:				
	3.3 Hz Fast Running	2.40 Hz 1st Natural Frequency	2.23 Hz 2nd Harmonics Excites 2nd Mode	2.81 Hz 2nd Harmonics Excites 3rd Mode	1.87 Hz 3rd Harmonics Excites 3rd Mode
CS1	0.06	0.20	0.15	0.06	0.07
CS2	0.05	0.24	0.15	0.07	0.05
CS3	0.04	0.32	0.12	0.08	0.07
CS4	0.06	0.36	0.16	0.09	0.06

**Table 10:** Maximum vertical accelerations generated by a single pedestrian running obtained at the representative points of the cable-stayed footbridge with the GFRP slab for the selected frequencies.

Point	Maximum Accelerations [m/s^2^] at Frequency:			
	3.3 Hz Fast Running	3.09 Hz 2nd Harmonics Excites 2nd Mode	2.06 Hz 3rd Harmonics Excites 2nd Mode	2.51 Hz 3rd Harmonics Excites 3rd Mode
CS1	0.32	0.51	0.44	0.28
CS2	0.31	0.38	0.40	0.17
CS3	0.46	0.35	0.32	0.27
CS4	0.59	0.38	0.24	0.19

**Table 11:** Maximum vertical accelerations generated by a single pedestrian walking obtained at the representative points of the beam footbridge with the RC slab the selected frequencies.

Point	Maximum Accelerations [m/s^2^] at Frequency:		
	2.4 Hz Fast Walking	2.00 Hz 1st Natural Frequency	1.37 Hz 2nd Harmonic Excites 2nd Mode
B1	0.01	0.05	0.03
B2	0.02	0.06	0.02
B3	0.01	0.05	0.03

**Table 12:** Maximum vertical accelerations generated by a single pedestrian walking obtained at the representative points of the beam footbridge with the GFRP slab for the selected frequencies.

Point	Maximum Accelerations [m/s^2^] at Frequency:			
	2.4 Hz Fast Walking	1.38 Hz 2nd Harmonics Excites 1st Mode	1.91 Hz 2nd Harmonics Excites 2nd Mode	1.64 Hz 3rd Harmonics Excites 3rd Mode
B1	0.09	0.09	0.26	0.19
B2	0.14	0.11	0.06	0.26
B3	0.10	0.10	0.26	0.21

**Table 13:** Maximum vertical accelerations generated by a single pedestrian running obtained at the representative points of the beam footbridge with the RC slab for the selected frequencies.

Point	Maximum Accelerations [m/s^2^] at Frequency:		
	3.3 Hz Fast Running	2.00 Hz 1st Natural Frequency	2.73 Hz 2nd Natural Frequency
B1	0.06	0.13	0.13
B2	0.08	0.18	0.03
B3	0.08	0.12	0.14

**Table 14:** Maximum vertical accelerations generated by a single pedestrian running obtained at the representative points of the beam footbridge with the GFRP slab for the selected frequencies.

Point	Maximum Accelerations [m/s^2^] at Frequency:			
	3.3 Hz Fast Running	2.76 Hz 1st Natural Frequency	1.91 Hz 2nd Harmonics Excites 2nd Mode	2.45 Hz 2nd Harmonics Excites 3rd Mode
B1	0.14	0.36	0.43	0.29
B2	0.13	0.48	0.08	0.46
B3	0.14	0.35	0.44	0.34

**Table 15:** The risk of resonance assessment for: the cable-stayed footbridge with the RC slab (Variant CS_RC), the cable-stayed footbridge with the GFRP slab (Variant CS_GFRP), the beam footbridge with the RC slab (Variant B_RC) and the GFRP slab (Variant B_GFRP).

Footbridge type_Slab Variant	No. of Vertical Mode	Frequency [Hz]	Risk of Resonance				Frequency Ranges in a Vertical Direction [Hz]
			Negligible	Low	Medium	Maximum	
							0.0–1.0
							1.0–1.7
B_RC ^1^	1st	2.00					1.7–2.1
CS_RC ^2^	1st	2.40					2.1–2.6
B_RC ^1^	2nd	2.73					2.6–5.0
B_GFRP ^3^	1st	2.76					
CS_GFRP ^4^	1st	3.45					
B_GFRP ^3^	2nd	3.83					
CS_RC ^2^	2nd	4.45					
B_GFRP ^3^	3rd	4.91					
CS_RC^2^	3rd	5.61					
CS_GFRP ^4^	2nd	6.17					
CS_GFRP^4^	3rd	7.53					
B_RC ^1^	3rd	7.78					>5.0

^1^ B_RC—the beam footbridge with the RC slab; ^2^ CS_RC—the cable-stayed footbridge with the RC slab; ^3^ B_GFRP—the beam footbridge with the GFRP slab; ^4^ CS_GFRP—the cable-stayed footbridge with the GFRP slab.

**Table 16:** Comparison of the maximum accelerations of the footbridges with the traditional RC or the innovative GFRP slab material.

Footbridge Slab Material	Maximum Vertical Acceleration [m/s^2^] Obtained for:			
	Cable-Stayed Footbridge		Beam Footbridge	
	Walking	Running	Walking	Running
RC	0.15	0.36	0.06	0.18
GFRP	0.21	0.59	0.26	0.48
GFRP/RC acceleration ratio	1.4	1.6	4.3	2.7

**Table 17:** Comparison of the maximum accelerations of the footbridges with the traditional RC or the innovative GFRP slab material.

Footbridge Slab Material	Modal Masses and Total Mass [t] of:							
	Cable-Stayed Footbridge				Beam Footbridge			
	m_1_	m_2_	m_3_	Total	m_1_	m_2_	m_3_	Total
RC	39.1	49.3	‘130.8	267.1	95.4	73.6	59.5	292.8
GFRP	14.5	20.0	34.6	188.7	16.1	12.2	21.8	69.0
RC/GFRP mass ratio	2.7	2.5	3.8	1.4	5.9	6.0	2.7	4.2
